# Recycling biofloc waste as novel protein source for crayfish with special reference to crayfish nutritional standards and growth trajectory

**DOI:** 10.1038/s41598-020-76692-0

**Published:** 2020-11-11

**Authors:** Roman Lunda, Koushik Roy, Petr Dvorak, Antonin Kouba, Jan Mraz

**Affiliations:** Faculty of Fisheries and Protection of Waters, South Bohemian Research Center of Aquaculture and Biodiversity of Hydrocenoses, University of South Bohemia in České Budějovice, Na Sádkách 1780, 370 05 Ceske Budejovice, Czech Republic

**Keywords:** Biochemistry, Biological techniques, Zoology

## Abstract

Screening of novel feedstuffs, that too for data-deficient (nutritionally) animals, is somewhat ambiguous or problematic. Through systematic meta-analyses, the present study formulated most up-to-date crayfish nutritional standards, against which a recyclable waste (biofloc biomass, BM) from intensive aquaculture systems was assessed as a novel protein source. Growth trajectory dependencies and thermal growth coefficient qualifying for good growth in crayfish (TGC 0.5–0.64 units) were benchmarked. Using these standards and a 7-week growth trial, BM's suitability as a novel protein source for red swamp crayfish *Procambarus clarkii* was evaluated through its graded inclusions in a commercial feed. Results suggest that BM can elevate growth at 33–66% inclusion in existing feed formulations. Beyond 66% inclusion, BM can deteriorate growth in crayfish due to high ash content (exceeding physiological limit > 14%), arginine deficiency (~ 14–20% lower than an optimum requirement), and insufficient non-protein energy: protein ratio (3.7 cal mg^−1^). Arginine is perhaps the most critical amino acid in dietary protein for crayfish, and deficient in BM. Although no critical bioaccumulation levels of heavy metals were breached by feeding 100% BM to crayfish, a mineral and heavy metal (Hg) stress seemed plausible. Crayfish raised solely on biofloc may not realize full growth potential.

## Introduction

Freshwater crayfish, mostly endemic to the continents of North America, Australia-Oceania, and Europe^1^, account for 1.71 million tons of global aquaculture production with a worth of 14.46 billion € as of 2018^2^. Presently they contribute a negligible fraction in the global aquaculture scenario (~ 3.5% of total freshwater aquaculture production) but having great potential ahead. During the last half-decade alone (2013–2018), freshwater crayfish production, and its commercial valuation have tripled^2^. In terms of crayfish nutrition research, efforts have been quite limited compared to other commercially important crustaceans (like penaeids and palaemonids)^3,4^. Therefore, screening of novel feedstuffs, that too for crayfish, is somewhat ambiguous or problematic. A brief prologue in this regard is provided in the supplementary text. On the other hand, aquaculture nutrition research has focused on developing feed substitution strategies with a minimal supply of fishmeal and fish oil in recent times. One potential ingredient could be a microbial biomass meal from biofloc technology systems (BFT)^4^. BFTs are a modern, intensive aquaculture system that evolved from the classic ‘activated-sludge based sewage bioremediation’ in wastewater treatment plants. The system essentially operates on the rationale of maintaining an optimum C: N ratio (6:1 to 15:1) by daily purging with carbohydrate (carbon) source^5,6^. It is done to support the blooming of microbial biomass (flocs). These microbial flocculants, known as ‘bioflocs’ bioremediate the nitrogenous wastes generated by fish and uneaten feed into consumable microbial protein for cultured animals^7,8^. Although they are consumed by the fish or shrimp stock, the biofloc biomass (as measured in Imhoff cones) or total suspended solids (TSS) may often exceed the recommended values for fish (25–50 ml L^−1^; TSS up to 1000 mg L^−1^) and shrimp (10–15 ml L^−1^; TSS 400–600 mg L^−1^)—posing problems for the cultured animals^7,9–11^. It is advisable to drain part of the biofloc biomass daily through sedimentation or fractionation of biofloc system water^10,12–14^. Such thinning (filtering) of culture water generates a large amount of biofloc biomass as waste, quite frequently. This drained biofloc is often of limited use. In general, they can be used as an alternative to synthetic polymers for wastewater treatment^15^, fertilizer, or inoculum to start a new system^16^.

Our research intervenes in recycling this waste for aquatic animal nutrition. Since conventional protein sources in aquafeed (e.g., fishmeal) are becoming expensive and scarce, there has been a growing impetus in testing biofloc as an unconventional protein source for aquatic animals^8,17–19^. Few commercial floc meals are generically marketed under ‘single-cell protein (SCP)’ or ‘microbial protein’ category—Profloc (Nutrinsic), FeedKind (Calysta), and Novacq/OBM (Ridley, Maritech) with pricing (as of 2018) between 1.1–3.3 USD kg^−1^^17,18^. One of these is listed in IAFFD (international aquaculture feed formulation database), with complete nutrient spectrum data, including essential amino acids^20^. So far, crayfish are not included in these mentioned researches. The novelty here is its potential use as a feedstuff (protein source) in the crayfish diet. In general, the protein (12–49%), lipid (0.5–12.5%), and ash (13–46%) contents in biofloc can vary substantially depending on several factors (reviewed by^22^). To the best of our knowledge, nutritional evaluation of biofloc as a feedstuff ingredient for artificial crayfish diets has not been done so far. Although rearing of crayfish in BFT system, where the animals co-fed on commercial feed pellets (primarily) and bioflocs suspended in the system, are recently being explored^23,24^. Our objective was to understand—(a) nutritional optima of freshwater crayfish from the available literature in the absence of centralized recommendations (see supplementary material); (b) growth trajectory and nutritional dependencies in crayfish (supplementary material); (c) response of red swamp crayfish to biofloc meal in their diet, in terms of nutrition, growth, and survivability; (d) the risk of heavy metals bioaccumulation or mineral stress in crayfish from feeding on biofloc, and; (e) evaluate nutritional strengths and bottlenecks associated with using biofloc meal in crayfish diet. The first two objectives (a and b) were rather a methodological and necessary step (placed in supplementary material) to the second part of our research related to the use of biofloc meal for crayfish (objectives c to e).

## Results and discussion

### Nutritional optima, growth trajectory, and nutritional dependencies of crayfish

Based on our meta-analyses, crayfish' optimum dietary nutritional requirement is tabulated as crayfish standards in Table 1. It is also compared with established standards of penaeid shrimps, often assumed as a template for most crustacean diets. Detailed information in this regard can be found in the supplementary material. In terms of crayfish growth trajectory, their thermal growth coefficient (TGC) may vary from 0.07–1 unit (interquartile range, IR 0.32–0.64 units). Results suggest any TGC in the range of 0.5–0.64 units may be regarded as ‘reasonably good growth’ in crayfish. Further insights into crayfish growth trajectory and its nutritional dependencies are presented in detail in the supplementary material. The information synthesized and approach used may serve as a template for future researchers exploring three less-established or unknown dimensions simultaneously (as in the present study)—novel feedstuff, optimum nutrition, and data-deficient (nutritionally) animals.

**Table 1:** Optimum dietary nutritional requirement of freshwater crayfish and its comparison with NRC (2011) standards for penaeid shrimps (usually adopted as *status quo*).

Parameter	Crayfish standard (calculated)	NRC^4^ standards for penaeid shrimps
**Macronutrient and energy (based on** ***Cherax*** **sp. and** *** Procambarus*** **sp.)—crude**		
Crude protein	29–34% (44%)*	33–42%**
Crude lipid	6.5–9%	5–6%
Crude NFE (nitrogen-free extract)	40–47%	–
Dietary fiber	Up to 7%	–
Total ash	7.8–10.8%	–
Gross energy	3590–4205 kcal kg^−1^	3666–4888 kcal kg^−1^**
Protein: Energy	72–91 mg kcal^−1^ (113–119 mg kcal^−1^)*	85–90 mg kcal^−1^
Non-protein energy: Protein ratio	5.3–8.5 cal mg^−1^ (4.4–4.8 cal mg^−1^)*	–
**Essential amino acids (based on** ***P. clarkii*** ** only)—digestible**		
Leucine	1.8–2.5%	1.8%
Valine	1.2–1.6%	1.4%
Threonine	0.3–1.5%	1.3%
Isoleucine	1.2–1.7%	1.2%
Arginine	**2.1–2.7%**	1.8%
Phenylalanine	0.8–1.5%	1.4%
Lysine	1.2–2.4%	1.8%
Methionine	1.1–4.9%	0.7%
Histidine	0.6–0.9%	0.7%
Tryptophan	0.4%	–
**Essential minerals (based on** ***Astacus*** **sp.,** ***Ornectes*** **sp., and** ***Procambarus*** **sp.)—available**		
Calcium	3000–4000 mg kg^−1^	–
Phosphorus	164–235 mg kg^−1^	3000–7000 mg kg^−1^
Iron	27–125 mg kg^−1^	–
Zinc	10–14 mg kg^−1^	15 mg kg^−1^
Copper	6–9 mg kg^−1^	10–32 mg kg^−1^
Manganese	14.2–17.8 mg kg^−1^	–

*In parentheses—proposed reconsideration of calculated standards, based on high TGC obtained in the present trial.

**Digestible values converted to crude values assuming 90% apparent digestibility.

### Growth response of crayfish to biofloc protein

**Figure 1:**
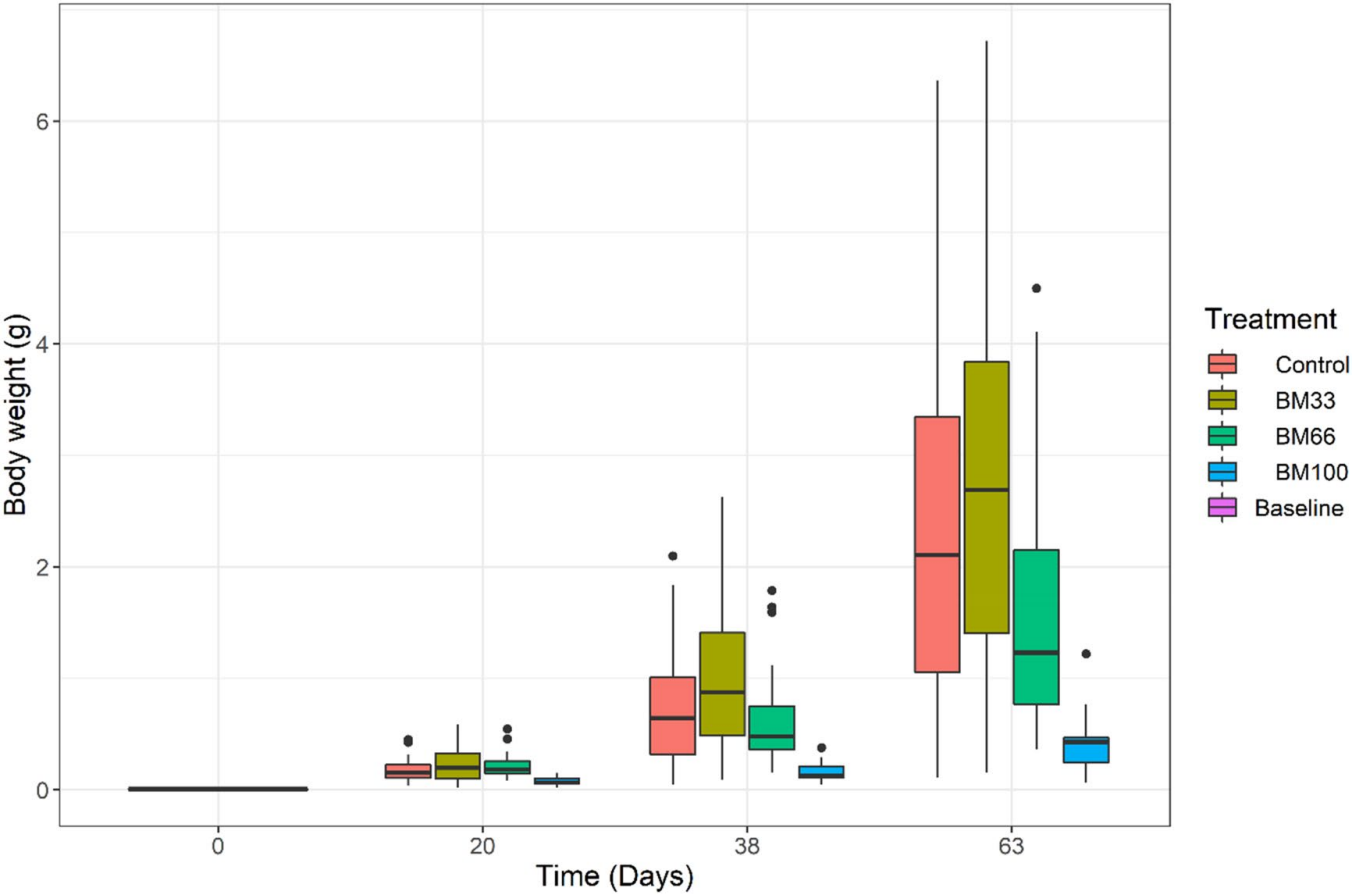
Body weight distribution in red swamp crayfish *Procambarus clarkii* fed graded level of biofloc meal (BM) in diets over 9 weeks of experimental duration. Measured on 20th, 38th and 63rd days post stocking. ‘Baseline’ indicates stocked stage-3 juveniles (0.007–0.008 g individual^−1^). Size heterogeneity (measured by coefficient of variance, CV) seems maximum and comparable in control (mean CV = 67%), BM_33_ (mean CV = 67.5%) and BM_66_ (mean CV = 63.4%) groups but significantly suppressed (*p* < *0.05*) in BM_100_ (mean CV = 51%). BM_100_ showed poor size throughout the experiment.

Following a 9-week growth trial with graded BM levels in the diet, differential growth response by crayfish was realized (Fig. 1). Except for control and BM_33_ groups, crayfish' final body weight showed a significant deviation from the normal distribution. Further examining the skewness of final body weight distribution in BM_66_ and BM_100_ groups, it was apparent that these groups were dominated by runts (smaller sized individuals) with large size deviations from the handful of bigger individuals. The size heterogeneity showed a significant and negative correlation with BM inclusion in the diet (Pearson's 2-tailed r =  − 0.63, *p* < *0.05*). Size heterogeneity in crayfish may aggravate community aggression^25^. However, the diet-driven size heterogeneity was not significantly correlated with mortality. The dietary treatments did not cause significant differences (*p* > *0.05*) in survivability, confirmed by *post-hoc* analyses. Overall, the survivability remained > 70% through the experimental period in all groups (Table 2). It implies—BM does not pose a significant mortality risk to crayfish stocks irrespective of inclusion levels, but it has implications on growth (presented below).

**Table 2:** Response of the red swamp crayfish *Procambarus clarkii* (initial body weight 7–8 mg) under 9-week growth trial (21.8 °C) fed experimental diets. Values presented in interquartile range with mean ± standard deviation in parentheses.

Diet group	Survival (%)	Final body weight (g)	Live weight gain (mg day^−1^)	Food conversion ratio	Protein efficiency ratio	Thermal growth coefficient
Control	70^a^	1.06–3.34 (2.44 ± 1.79)^a^	17–53 (39 ± 15)^a^	1.2*	2	0.60–0.94 (0.84 ± 0.14)^a^
BM_33_	70^a^	1.40–3.84 (2.80 ± 1.86)^a^	22–61 (44 ± 16)^a^	1.4	1.6	0.68–0.99 (0.89 ± 0.13)^a^
BM_66_	80^a^	0.77–2.15 (1.62 ± 1.19)^a^	12–34 (26 ± 9)^a^	1.5	1.5	0.53–0.79 (0.72 ± 0.11)^a^
BM_100_	83^a^	0.25–0.47 (0.41 ± 0.25)^b^	4–7 (6 ± 1)^b^	1.8*	1.3	0.32–0.42** (0.40 ± 0.04)^b^

^a,^^b^Superscripts denote statistically different (*p* < *0.05*) groups.

*Pattern: FCR multiplied by Arginine content of feeds ≈ fulfillment of Arginine requirement (as per crayfish or penaeid standards).

**Below reasonably good growth (TGC 0.47–0.59) for crayfish standards.

**Figure 2:**
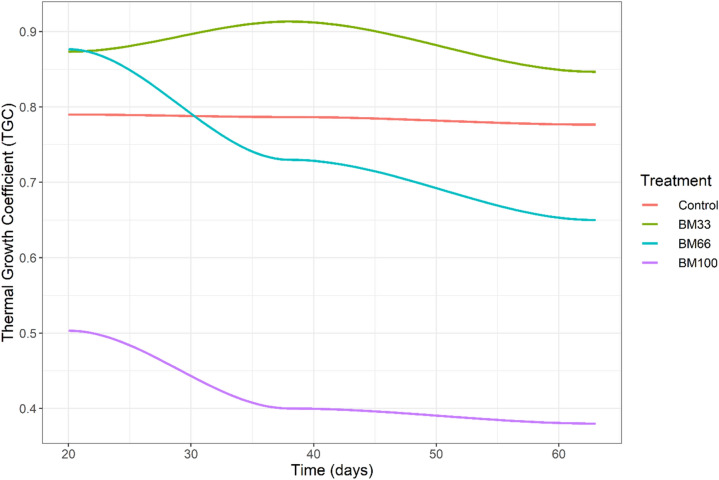
Growth pattern (TGC: thermal growth coefficient) of red swamp crayfish *Procambarus clarkii* fed different experimental diets over 9 weeks. A dampening of growth over time gradually setting-in at higher BM inclusion in the crayfish diet (from BM_66_ to BM_100_). At the end of culture, BM_100_ resulted in twice less growth (*p* < *0.05*) than achievable on other diets (control or BM_33_ and BM_66_—statistically comparable TGC).

**Figure 3:**
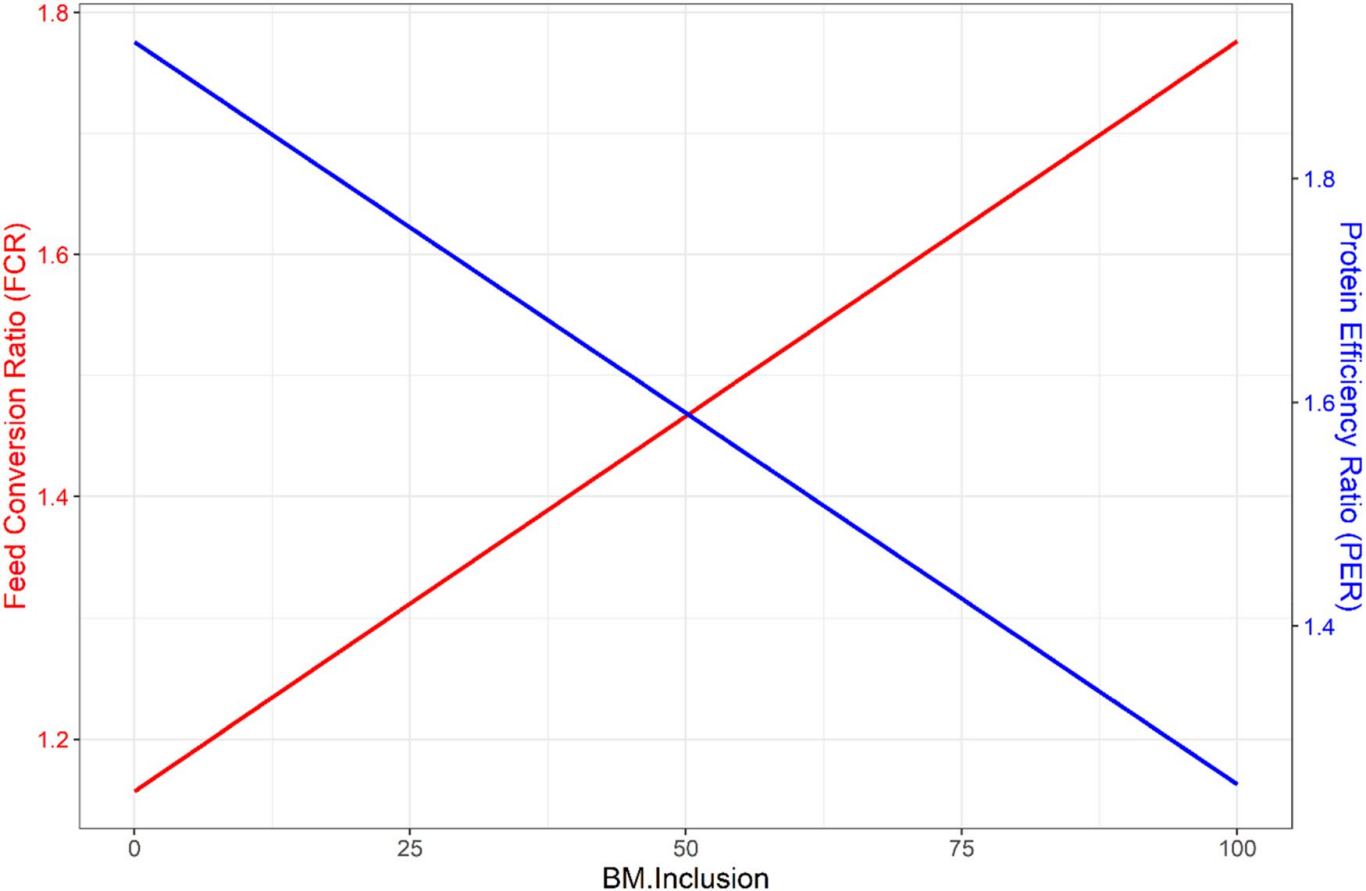
Feed utilization pattern (FCR in red and PER in blue) of red swamp crayfish *Procambarus clarkii* in response to the level of biofloc meal (indicated by BM.Inclusion, in %) in the diet. More feed is required per unit weight gain of crayfish with an increasing share of BM in the diet because protein utilization is lowered at higher BM inclusion.

The growth in terms of TGC, live-weight gain (LWG), and body weight (BW) were significantly depressed (*p* < *0.05*) in the BM_100_ fed group. In contrast, the growth in control, BM_33,_ and BM_66_ groups were higher with insignificant differences among them (*p* > *0.05*) (Table 2, Fig. 1, 2). A statistically insignificant dampening of growth rate over time (*p* > *0.05*) was observed in groups BM_66_ and BM_100_ (Fig. 2). At the end of culture (63 days), the realized TGC in crayfish fed on BM_100_ was on an average two times lower (*p* < *0.*05) than the growth exhibited on control, BM_33,_ or BM_66_ diets (Table 2, Fig. 2). In terms of feed utilization, the feed conversion ratio (FCR) and protein efficiency ratio (PER) were linearly related to increasing BM inclusion in the diet. The FCR increased with increasing share of BM in diet**:** FCR = 1.156 + 0.006 × BM (Adj. R^2^ 0.95, *p* < *0.05*). The PER decreased with an increasing BM inclusion: PER = 1.922 − 0.006 × BM (Adj. R^2^ 0.95, *p* < *0.05*). It means, for every 10% inclusion of BM, FCR increased by + 0.06 units, and PER decreased by − 0.066 units (Fig. 3). The results from the growth trial are summarized in Table 2, and the relationship of feed utilization parameters in response to BM inclusion is depicted in Fig. 3. Interestingly, the calculated FCR(s) of our respective diets, when multiplied with the dietary arginine content, seem to ‘hit the target’ of arginine requirements by crayfish (e.g., FCR of BM_100_ × Arginine in BM_100_ = Fulfillment of arginine requirement).

As per the crayfish growth trajectory (quantified in the previous section), the group fed on 100% BM failed to show reasonably good growth. They were dominated by smaller-sized runts, poorest of the FCR and PER, but no significant mortality. Among the limited studies testing flocculated microbial meals in crustacean diets [reviewed in 8], BM inclusions were mostly up to 10–30% (of the total diet) or 30% (of fishmeal replacement). Good results in terms of growth were usually obtained at the maximum inclusion levels^8^. Like the present study, two previous studies had tested BM (on *Litopenaeus vannamei*) at a broader inclusion level from 17 to 84% of the total diet^17,26^. Despite different target species, the results seem close to that of the present study. Above 41–53% BM inclusion, the growth advantages were gradually lost^17,26^. Looking deeper into the aspects of our BM_100_-protein compared to control, BM_33_, or BM_66_-protein, the arginine seems to be a bottleneck for reasonably good growth (Tables 2, 4). Other EAAs, which could also be critical (e.g., methionine and lysine), were comparable-to-higher in BM_100_ than in other diets (Table 4). Although methionine and lysine levels in diets fell short of our formulated crayfish nutritional standard (Table 1), at least it fulfilled penaeid EAA standards of NRC^4^. It hints that NRC’s penaeid EAA standards cover well for most of the EAA requirements in crayfish, except for arginine (and tryptophan could not be judged). Arginine levels in BM (Table 4) neither fulfilled crayfish nor penaeid standards (Table 1).

Biofloc has been previously criticized for being partly deficient in arginine^27–29^. The arginine coefficient (proportion of total protein, in %) of biofloc meals, be it commercial ones like Novacq (2.38%^19^), FeedKind (2.54%^20^), or in the present study (2.73%) seem to have close resemblance (CV 5.5%). If we consider the mean arginine coefficient of BM from these data (2.55%) and tally it to fulfill the optimum arginine requirement of crayfish (minimum 1.8%), the crude protein level of such BM should be at least 70%. It is beyond the expected range of ordinary bioflocs^22^. BM harvested from high TSS systems (due to infrequent sedimentation or water exchange) can have lower protein content^10^. For example, the crude protein content of a biofloc can drop by − 34.5% if the TSS of the system is let to increase from ≤ 200 mg L^−1^ to 800–1000 mg L^−1^^10^. As such, BM harvested from a low TSS system would have higher arginine (0.72%) compared to a high TSS system (0.47% arginine) (recalculated from^10^; using mean arginine coefficient = 2.55% of total protein). Even with aging biofloc, the content of arginine (also other EAAs) may decline. For example, from the 10th day to the 30th day of a biofloc culture, the arginine levels can decrease by 25–41% (recalculated from^27^). However, some specially produced commercial flocculated meals can have a high arginine coefficient (e.g., 5.3% of the protein in ProFloc^17^). Among all the EAAs, arginine content in red swamp crayfish seems maximum^3,21,30,31^, indicating a supposedly higher arginine demand in crayfish. The same is true for marbled crayfish *Procambarus virginalis*^32^. Arginine is perhaps the most limiting EAA in most crustacean diets and is required between 1.6–2.7% of diet^33^. Due to the poor activity of the urea cycle in crustaceans, arginine is indispensable for growth^33,34^. Arginine functions as a phosphagen in crustaceans, being the only amino acid providing amidino group for the synthesis of creatine—a major reserve of high-energy phosphate for ATP regeneration^33,35^.

### Risk of heavy metals bioaccumulation or mineral stress from biofloc meal

The contents of heavy metals in BM were below the critical pollution limits. No critical limits were breached in the crayfish body that could qualify BM as a feedstuff capable of inducing unsafe heavy metal biomagnification, rendering them unfit for consumption. Content of Cd and Mn were mostly below the detection limits (Table 3). Except for mercury, hepatopancreas contained a higher amount of heavy metals (and minerals) than muscle. Hepatopancreas of crayfish, like most crustaceans, have been reported to be major storage of minerals, including heavy metals^3,37^. With increasing BM fraction in the diet, the concentration of Hg significantly increased in hepatopancreas (control → BM_33_ and BM_66_ → BM_100_; *p* < 0.05), while other metals did not show any significant trend (Table 3). Except for Cd, all metals were significantly higher (*p* < 0.05) in the hepatopancreas of BM_100_ fed crayfish compared to the control group. Such accumulation of heavy metal in hepatopancreas is capable of impairing metabolism in crayfish^37^. The concentration of Fe exhibits a rather ‘bell curve’ pattern, peaking at BM_33_ and receding thereafter, only in the muscle (Table 3). Cd and Zn did not exhibit any pattern as such. The heavy metal contents in crayfish and BM are given in Tables 3 and 4, respectively.

**Table 3:** Heavy metals and mineral content (mean ± SE; dry matter basis) in the tail muscle and hepatopancreas of red swamp crayfish *Procambarus clarkii* fed graded levels of biofloc meal.

Group	Hg (µg kg^-1^)	Mn (mg kg^-1^)	Cd (mg kg^-1^)	Zn (mg kg^-1^)	Fe (mg kg^-1^)
**Muscle**					
Control	9.4 ± 0.9^a^	BDL	0.008 ± 0.01^a^	11.4 ± 1.4^a^	4.1 ± 2.7^a^
BM_33_	10.5 ± 1^b^	BDL	BDL	11.8 ± 0.9^a^	7.0 ± 5.7^b^
BM_66_	10.4 ± 1.4^b^	BDL	BDL	10.4 ± 1.0^b^	3.2 ± 2.4^a^
BM_100_	12.8 ± 1.2^c^	BDL	BDL	8.5 ± 0.5^c^	BDL
**Hepatopancreas**					
Control	4.6 ± 1.0^a^	2.2 ± 0.1^a^	0.17 ± 0.05^a^	46.1 ± 30.0^a^	54.6 ± 13.0^a^
BM_33_	5.4 ± 0.6^b^	2.9 ± 0.4^ab^	0.13 ± 0.03^b^	72.4 ± 26.3^b^	90.4 ± 13.4^b^
BM_66_	5.4 ± 0.7^b^	3.2 ± 0.8^b^	0.13 ± 0.01^b^	67.7 ± 34.8^ab^	88.0 ± 6.0^b^
BM_100_	11.0 ± 1.2^c^	3.6 ± 2.2^b^	0.19 ± 0.01^a^	76.3 ± 28.9^b^	82.4 ± 12.0^b^

BDL = below detection limit (Mn and Fe: < 2 mg kg^-1^, Cd: < 0.002 mg kg^-1^); different letters in superscript denote groups with significant differences as derived from Tukey’s HSD multiple range test (α = 0.05).

**Table 4:** Proximate composition of biofloc meal, basal and treatment diets (dry matter basis).

Proximate fraction	Basal	BM_33_	BM_66_	BM_100_
Crude protein (CP) (%)	44.2	44.1	44	43.9
Crude lipid (%)	7.8	6.7	5.6	4.5^a^
Crude NFE (%)	35.5	33.8	32.1	30.3
Crude Fibre (%)	2.7	3.4	4.2	4.9
Total Ash (%)	9.8	12	14.2	16.4*
Gross energy (kcal kg^−1^)	3890	3719	3549	3373
Protein: Energy ratio (mg kcal^−1^)	113.6	118.6	124	130.2
Non-protein energy: Protein ratio (cal mg^−1^)	4.8	4.4	4.1	3.7*
**Essential amino acids (%)**				
Leucine	2.3	2.3	2.2	2.2
Valine	1.3	1.5	1.6	1.8
Threonine	1.1	1.3	1.4	1.6
Isoleucine	1	1.1	1.1	1.2
Arginine	1.5	1.4	1.3	1.2**
Phenylalanine	1.4	1.5	1.7	1.8
Lysine	1.7	1.7	1.8	1.8
Methionine	0.6	0.6	0.7	0.7
Histidine	0.8	0.8	0.8	0.8
Tryptophan	–	–	–	–
**Minerals and heavy metals (mg kg**^**-1**^**)**				
Arsenic (As)	< 0.21	< 0.21	< 0.21	< 0.21
Cadmium (Cd)	0.41	0.6	0.7	0.90
Chromium (Cr)	2.06	3.9	5.8	7.72
Copper (Cu)	11.70	110.1	208.6	310^#^
Iron (Fe)	185	2437.3	4689.5	7010^#^
Mercury (Hg)	0.01	0.03	0.04	0.06
Manganese (Mn)	59.60	220.4	381.3	547^#^
Nickel (Ni)	2.06	4.2	6.4	8.67
Lead (Pb)	2.06	3.5	4.9	6.32
Zinc (Zn)	93.30	306.4	519.5	739^#^

*Matching the values with crayfish standards (Table 1)—hints under-supply (lipid, NPE:P) or excessive supply (ash).

**Matching the values with crayfish standards (Table 1) and optimistic assumption of biofloc protein digestibility (~ 90%)—hints under-supply of amino acid.

^#^Matching the values with crayfish standards (Table 1) and most conservative assumption of mineral retention (~ 10% retention)—hints mineral stress due to over-supply.

Globally, the total ash content in biofloc may range between 13–46% (reviewed by^22^), also applicable in our case. The problem of high ash content in most biofloc, limiting its inclusion in diets (despite good protein content), has been briefly discussed in Sabry Neto et al.^38^. One previous study, which studied BM at a high enough inclusion level, attributed high ash and probable toxic effects of trace minerals to retarded growth in *Litopenaeus vannamei* fed > 60% BM in a diet^26^. Owing to high ash content in BM, mineral stress seems plausible in the present study as well (*see* Tables 1, 4). By mineral stress, we imply even if 10% of the ash or minerals from BM are digested by crayfish, it is potentially much higher ‘bioavailable minerals’ in the body than their optimum physiological limits. Information on this aspect have been limited for shrimps [reviewed in 39, 40] and none for crayfish^3,36^. In shrimps (*Penaeus monodon, P. japonicus*), retarded growth was observed when excessive mineral premixes were supplemented in a practical diet^39^, or more specifically, when trace minerals like Fe and Mn exceeded levels of 0.01% each in the diet^40^. The BM_100_ had all these factors (ash, Fe, and Mn) in excess (Table 4). Heavy metal stress could also be plausible. Any significant absorption of Hg in the body (presented above) is capable of impairing crayfish metabolism^37^, provoking hyper-osmoregulation in crustaceans^41^, with repercussions on aggravated energy expenditure^42^. Our metadata derived models show TGC in crayfish deteriorates at dietary ash levels > 14% (also in BM_100_), during which the retention of ash is merely < 10% of total dietary intake (see supplementary material and Fig S2, S3). Thus ≥ 90% of the ingested ash (exceeding physiological limits) are excreted through digestive and osmoregulatory (metabolic) pathways. It has its own energy cost, which could have been utilized for protein-sparing or growth^42^.

### Recycling biofloc waste as a novel feedstuff for crayfish: Strengths and bottlenecks

Comparing the nutritional standards for crayfish with observed performance in growth trials, few strengths and bottlenecks of BM were realized (Tables 1 and 4). In terms of advantages: (a) BM has a high crude protein content (43.9%); (b) crude fiber content in BM (4.9%) was in the optimum range for crayfish, and; (c) BM is a rich supplier of minerals. However, there are more bottlenecks than limited advantages. BM has excessive total ash detrimental to crayfish growth, with probable manifestations on hyper-osmoregulation and energy expenditure (discussed above). A mediocre crude lipid content (4.5%) is another bottleneck for supplying non-protein energy. These, in combination, render the non-protein energy: protein ratio (NPE: *P* = 3.7 cal non-protein energy per 1 mg protein) in BM insufficient for effective protein sparing (≈growth). At such low NPE:P, the proteins are catabolized for meeting energy demand (even after oxidizing carbohydrates and lipids), rather than building biomass^42^. It is further compounded by arginine deficiency in BM (~ 14–20% less than an optimum requirement)—probably the most critical essential amino acid for crayfish (discussed above).

A retrospective evaluation of BM_100_ or BM (as a feedstuff for crayfish) applying our metadata derived ‘growth-retention models’ (supplementary Fig S3, S4, S6) could explain few nutrient utilization scenarios behind low growth in BM_100_. The ash, protein, and lipid retentions from BM should be less than 5%, 10%, and 3% of dietary intakes, respectively (predicted). For control, BM_33,_ and BM_66_ diets, these retentions were well above the identified thresholds qualifying for reasonably good growth in crayfish (refer to supplementary material). Comprehensively, the retarded growth problem with solely feeding on biofloc biomass could be a synergistic effect of— (a) arginine deficiency, (b) mineral and heavy metal stress, and, (c) low non-protein energy to protein ratio.

## Methods

### Calculation of crayfish nutritional standards, growth trajectory, and its nutritional dependencies

In the absence of centralized nutrition recommendations for freshwater crayfish species, unlike other commercially important crustaceans (e.g., penaeid shrimps, *see* NRC^4^), available literature was meta-analyzed. Peer-reviewed and published articles (in English or at least with English abstract) were searched online (search engines: Web of Science, Scopus, and Google Scholar) using keywords like ‘growth trials’, ‘crayfish’, ‘nutrition’, ‘proximate composition’, ‘body composition’, ‘amino acids’, ‘heavy metals’, ‘optimum requirement’ were used in different combinations (depending on target information). Altogether 27 articles were sourced and data extracted for meta-analyses. Detailed methodology on each meta-analysis (*i.e.,* formulation of nutritional standards, calculation of growth trajectory and feed utilization parameters, quantification of nutritional dependencies on growth) are provided in the supplementary material.

### Collection of biofloc biomass

Biofloc biomass was obtained from a well-established indoor, freshwater biofloc system, stocked with Nile tilapia *Oreochromis niloticus* at a stocking density of 35 kg m^−3^. Commercial pellets (TILAPICO 3 mm, Coppens, The Netherlands) were used as standard feed for fish. Fish feed was given twice daily based on a feed amount equivalent to 2.5% of the fish body weight. Wheat flour (35.56% C; 2.38% N) served as a carbon source which was applied daily with feed (22.05% C; 7.07% N) in a ratio of 1:0.6 (feed: flour). Assuming a 30% retention of nutrients from feed to fish, the projected C: N ratio was ≈6:1. Such a low C: N ratio favored frequent harvest of young and N-rich wet biofloc biomass^6,10^ to be converted to dry matter for the ensuing experiment. Biofloc biomass was drained daily through a pump and a vortex separation device so that the suspended solids level stayed between 25 and 50 ml L^−1^ in the system. After separation, biofloc was filtered through a nylon screen (mesh size 60 μm) to drain the excess water. The filtrate was then dried at 80 °C to obtain a material of solid consistency. After obtaining enough dried biofloc, the samples were grounded by a hammer mill to yield finer particles and hereinafter referred to as the biofloc meal (BM).

### Preparation of experimental feed

Commercial pellets (TILAPICO 3 mm, Coppens, The Netherlands) were used as the basal diet due to its similar protein content with our test ingredient (BM). The commercial ‘fish feed’ was chosen due to a lack of established ‘crayfish feeds’ in the market. Even the available ones appeared to be random feed mixtures targeted for ornamental crayfish keeping. Inclusion of BM by replacing basal diet was done on a weight by weight basis. All feeds were isonitrogenous. The graded inclusion levels were 0% (basal diet = control diet), 33% (67% basal + 33% BM; diet BM_33_), 66% (34% basal + 66% BM; diet BM_66_) and 100% (only BM; diet BM_100_). Feed pellets (pellet size 2 mm) were cold extruded, dried (12 h; 45 °C), vacuum sealed, and stored at 4 °C till further use. The diet samples were analyzed in an accredited third-party laboratory (AGRO-LA, spol. s.r.o., https://www.agrola.cz/zemedelske-a-potravinarske-sluzby/) employing analytical methods (ISO verified and certified protocols in the Czech Republic) for proximate composition, essential amino acids (EAAs; except tryptophan due to analytical error), heavy metals, and essential mineral contents. Detailed composition of basal diet, treatment diets and the biofloc meal are summarized in Table 4.

### Crayfish keeping

A total of 120 juvenile red swamp crayfish (*Procambarus clarkii;* conservation status: least concern) having a mean weight of 7.8 ± 0.7 mg at the onset of exogenous feeding (developmental stage 3), were used as experimental animals (10 individuals per tank; 4 group x triplicate). The experiment lasting for nine weeks was conducted in a series of indoor glass aquaria (54 × 36 × 30 cm, volume 46 L) with aeration and attached to a recirculating aquaculture system. Two baked clay bricks (28.5 × 13.5 × 6.5 cm), each with 39 cross holes (26 and 13 holes with a profile of 1 × 3 cm and 1 × 1 cm, respectively), were placed in each aquarium to provide shelters/refugia for the stocked crayfish^43^. After three weeks, a block of joined polypropylene tubes containing five tubes (length 10 cm, inner diameter 35 mm) was added to each aquarium as an additional shelter for on-growing animals. The bases were represented by three longitudinally joined tubes with a further two tubes positioned pyramidal in the second layer^44^. Altogether, 12 tanks were used and subjected to stable indoor climatic conditions with natural photoperiod (12L:12D).

### Growth trial and feed utilization parameters

Crayfish were fed twice a day to apparent satiation (roughly corresponding 5–6% of the body weight) with the abovementioned diets for nine weeks. Uneaten feed, feces, and other wastes were siphoned out manually every morning. Dissolved oxygen (7.9 ± 0.3 mg L^−1^), pH (7.6 ± 0.2), and temperature (21.8 ± 0.3 °C) were measured daily using Oxi 3205 and pH 720 m (WTW GmbH, Weilheim, Germany), respectively. Every three weeks, the body weight was measured using an electronic balance (lowest sensitivity 1 mg) and the number of survivors counted. The feed rationing was revised accordingly. Body weight measurements were taken before feeding. After the trial, final body weight and total length were recorded, including the number of survivors. The animals were not fed before the day of the final measurement.

The food conversion ratio (FCR, units), protein efficiency ratio (PER, units), and survivability (%) were determined for each diet following the formulas in Cortes-Jacinto et al.^45^. Live weight gain (LWG) was calculated applying the formula, LWG = final—initial weight (in mg)/ days reared. Coefficient of variance (CV) of body weight (standard deviation × 100 ∕ mean) was calculated as a measure of size heterogeneity. To eliminate statistical biasedness in the data due to hierarchical size distribution in crayfish groups, other measures of central dispersion like interquartile range (IR) and median were included besides the mean. The abovementioned parameters were calculated from the IR, median, and mean estimates of each treatment. All graphical models were generated using the ggplot2 package in R. Statistically significant differences (α level set at 0.05) in body weight, growth, and survivability of crayfish fed on different dietary treatments were tested. The grouped data were first subjected to a Shapiro–Wilk’s normality test; then following the *p value*, either one-way ANOVA with *post-hoc* Tukey HSD (parametric test), or, Kruskal–Wallis *post-hoc* Dunn’s test with Bonferroni correction (non-parametric test) was selected. The tests were performed using default commands in RStudio v1.2.5042.

### Assessment of heavy metals risk from biofloc biomass

At the end of the experiment, tail muscle and hepatopancreas samples from representative crayfish of each group were collected and frozen (−20 °C). Selected heavy metals (Hg, Cd, Zn; following high bioaccumulation affinity realized in Kouba et al.^46^) and some additional minerals (Fe, Mn) were analyzed from these samples in the same accredited third-party laboratory. Body (muscle + hepatopancreas) heavy metal levels were compared with maximum permissible limits (Cd or Hg 0.5 mg kg^−1^ wet weight basis) given in the European Commission^47^ for aquatic meat products (in the context of safety for consumption). In the context of agricultural use safety (as fertilizers), the heavy metal content of biofloc meal was determined and compared with Czech EPA limits (Cd 5 mg kg^−1^, Hg 4 mg kg^−1^ dry matter basis) (Decree of Ministry of Environmental of the Czech Republic No. 437/2016 on the Code, 2016).

### Ethics approval

All procedures performed in studies involving animals (*Oreochromis niloticus* and *Procambarus clarkii*) were in accordance with the ethical standards approved by the institutional ethics committee (Jihočeská univerzita v Českých Budějovicích Fakulta rybářství a ochrany vod).

## Supplementary information

Supplementary Information.

## Abbreviations

BM: Biofloc meal (biomass)
BFT: Biofloc technology aquaculture system
TGC: Thermal growth coefficient
EAA: Essential amino acid
IR: Interquartile range
GAM: Generalized additive model
LWG: Live-weight gain
CP: Crude protein
CL: Crude lipid
