# Supplementary material for: Recycling biofloc waste as novel protein source for crayfish with special reference to crayfish nutritional standards and growth trajectory
Source: Sci Rep. 2020 Nov 11;10:19607. doi: 10.1038/s41598-020-76692-0 (PMC7658255; doi:10.1038/s41598-020-76692-0)
Supplement: Supplementary file 1 — Supplementary Information. [file 41598_2020_76692_MOESM1_ESM.docx]

*Full Title*:

**Recycling biofloc waste as novel protein source for crayfish with special reference to crayfish nutritional standards and growth trajectory**

*Short title:*

**Biofloc and crayfish**

*Author list:*

Roman Lunda^#^ (ORCID: 0000-0002-0437-585X), Koushik Roy^#^ (ORCID: 0000-0001-7205-9034), Petr Dvorak, Antonin Kouba (ORCID: 0000-0001-8118-8612), Jan Mraz* (ORCID: 0000-0003-3545-1269)

*Authorship declaration:*

**^#^**RL and KR contributed equally to the work.

*Affiliation:*

University of South Bohemia in České Budějovice, Faculty of Fisheries and Protection of Waters, South Bohemian Research Center of Aquaculture and Biodiversity of Hydrocenoses, České Budějovice 370 05, Czech Republic.

**Address of Correspondence:*

Dr. Jan Mraz

Institute of Aquaculture and Protection of Waters

Faculty of Fisheries and Protection of Waters

University of South Bohemia in České Budějovice

Na Sádkách 1780, České Budějovice 370 05, Czech Republic.

Correspondence Email: [jmraz@frov.jcu.cz](mailto:jmraz@frov.jcu.cz)

**Introduction**

In terms of crayfish nutrition research, efforts have been quite limited in contrast to other commercially important crustaceans (like penaeids and palaemonids) [1]. Existing efforts are largely focused on optimizing protein, lipid and protein: energy ratio in artificial diets (to maximize growth), alternative protein sources, replacement of animal protein (mostly fish meal) with vegetable protein, etc. (reviewed in [2, 3]). Yet, there are no nutrition recommendations specifically outlined for freshwater crayfish [4]. Therefore, screening of novel feedstuffs, that too for crayfish, is somewhat ambiguous or problematic. In the present effort, we have dealt with such bottleneck by formulating ‘crayfish nutritional standards’, by meta-analyzing the available literature on crayfish nutrition (elaborated below).

**Results and Discussion**

***Formulation of crayfish nutritional standards***

The optimum dietary nutritional requirement of crayfish is tabulated (as crayfish standards, see main document Table 1) and compared with established standards of penaeid shrimps often assumed as template for most crustacean diets. The comparison with penaeid nutritional standards revealed: (a) protein requirement of crayfish is lower, but it is probably under-estimated (*i.e.* it should be comparable to penaeid shrimps; discussed later); (b) dietary lipid requirement in crayfish may be slightly higher; (c) there is overlap in gross energy requirements and protein-energy balance (or even slightly higher P:E ratio; discussed later); (d) essential amino acids (EAA) requirement of penaeids cover crayfish requirements well enough, except for arginine and methionine (arginine perhaps most critical one; discussed later); (e) mineral requirement in crayfish is somewhat lower-to-comparable (lack of sufficient data for comparison). Dedicated standards for crayfish aquaculture nutrition is still non-existent. Since the pioneering efforts by D’Abramo and Robinson [1] and Saoud et al. [2] to review crayfish nutritional requirements, there has been a paucity of such attempts. Existing crustacean standards, be it penaeids or palaemonids nutritional requirements given in NRC [4], are assumed as good proxies for crayfish. We systematically reviewed the existing literature through a meta-analytic approach and formulated the most up-to-date crayfish nutritional standards. The overlaps and shortcomings of penaeid nutritional standards (most standardized among all cultured crustaceans, [4]) to sufficiently address crayfish nutrition was also evaluated. To the best of our knowledge, this is the first such attempt.

In general, the EAA and protein standards of penaeids can cover freshwater crayfish requirements except for arginine. Arginine is perhaps the most critical EAA in crustacean diets, also demonstrated in the present study and elaborated below. A prologue on the importance of arginine especially in crustacean diets can be found in Zhou et al. [5]. So far, only lysine (1.6%) and methionine (0.9%) requirements have been studied for red swamp crayfish *P. clarkii* [6, 7]. Since only one study per EAA is available, it is difficult to validate these estimates at par our formulated standards. These estimates, however, fall between the range of penaeid EAA requirement given in NRC [4] and our formulated standards. Metadata simulated models (presented below) and high thermal growth coefficient (TGC) realized on our experimental diets (with 44% CP; see main document Table 2) hint following possibilities of revising crayfish protein requirement: (a) protein optima recommended in Saoud et al. [2] and proposed in some growth trials may be potentially under-estimated; (b) protein demand of crayfish may be somewhat similar-to-higher compared to shrimps. Also, the optimum protein-energy balance (P:E ratio) for crayfish is probably higher than penaeid standards. This is supported by Hubbard et al. [8] and the present study, with optimum P:E ratio being 100-119 mg kcal^−1^ (*i.e.* above the penaeid standards). To realize high growth potential in crayfish, protein retention >14% must be ensured by feeding diets with at least 31% crude protein (with sufficient EAAs, especially arginine) and balanced P:E ratio of about 100-119 mg kcal^−1^. Contradictions exist regarding lipid requirement in crayfish diet. The lipid (or non-protein energy) requirement in crayfish seem higher than penaeids [4, present study]. Davis and Robinson [9] argued against prioritizing lipids as an important macronutrient in crayfish diets. The present metadata analyses partly support this argument; lipid did not (statistically) contribute to TGC. While other studies showed lipids optimizing growth in crayfish at fixed protein levels [10, 11]. Albeit the controversial role of dietary lipids in crayfish diet, a nominal 5% lipid retention will most likely help in achieving good growth (present study). The dietary mineral requirement in crayfish or crustaceans *per se* is not very strict, if the water (habitat) has enough concentrations of these minerals [1]. For example – a minimum water hardness (proxy for Ca, Mg) of 17 mg L^−1^ (optimum 100 mg L^−1^) is important for the survival of red swamp crayfish (reviewed in [1]). It should be noted that some minerals (like P) cannot be readily absorbed from the water due to too low concentrations [1], and hence needed to be supplied through diet. However, too high feed ash levels (>14%) can deteriorate growth, probably by causing mineral stress that is manifested in reduced ash retentions (<16%).

***Growth trajectory and nutritional dependencies in crayfish***

The TGC in crayfish may vary from 0.07-1 unit (inter-quartile range, IR: 0.32-0.64 units). TGC in the range of 0.5-0.64 units (*i.e.* semi-upper IR) may be regarded as reasonably good growth in crayfish. Generalized additive model (GAM) on TGC-body size revealed two distinct growth stanzas (or, phases) in crayfish *(Adj. R^2^ 0.17, p<0.01.)*: stanza-A (1-14.5 g) and stanza-B (above 14.5 g) (Fig S1). Up to 14.5 g body size the growth in crayfish remain elevated (p>0.05), but it progressively slows down (p<0.05) beyond this point. Our experimental animals fell within the first growth stanza (*i.e.* the phase with highest growth potential). The b-value in length-weight relationship of our experimental animals (pooled) were suggestive of allometric growth (log_10_Weight= −8.3+2.9×log_10_Length; b ≈ 3, *Adj. R^2^ 0.878, p<0.01*).

The TGC metric is superior to the commonly used specific growth rate (SGR) metric [12]. To the best of our knowledge, meta-analyses on growth trajectory summarized for the first time the expected growth in freshwater crayfish (TGC from 0.07 to 1 units) and benchmarked TGCs to qualify for ‘reasonably good growth’ (=0.5-0.64 units). The growth trajectory identified in crayfish follows the typical growth phase (identified as stanzas [12]) as in Pacific whiteleg shrimp *Litopenaeus vannamei* [12]. It is characterized by high growth potential in smaller individuals (up to 14.5 g) but slowing growth with increasing body size (above 14.5 g). This is supported by the fact that most crustaceans have a finite size and grow toward an asymptotic weight [13], which was also visualized by our metadata-derived model (Fig S1). Understanding the growth trajectory in crayfish may aid in their efficient aquaculture, e.g. predicting final body size, proper feeding and nutrition decisions, realization or non-realization of full growth potential, harvesting decisions, etc. [12].


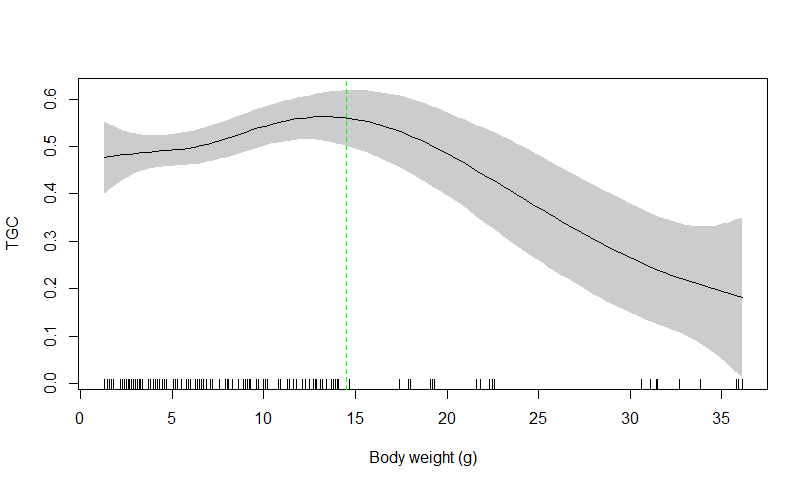


**B**

**A**

**Fig S1: Body size dependent growth trajectory of crayfish**. Divided into two growth stanzas A (left; elevated growth) and B (right; slowing growth) at cut-off body weight of 14.5 g.

In terms of nutritional dependencies (in descending order of importance): ash retention > feed ash > protein retention > feed crude protein > lipid retentions were identified as key determinants of TGC (*multiple R^2^ 0.819, p<0.01*) irrespective of growth stages. Individual GAMs (models) of TGC vs. feed ash (*Adj. R^2^ 0.249, p<0.05*) and TGC vs. ash retention (*Adj. R^2^ 0.875, p<0.05*) revealed good growth in crayfish at ash retentions >16% of dietary intake and feed ash levels below 14% (Fig S2, S3). Models of TGC vs. protein retention (*Adj. R^2^ 0.758, p<0.05*) and TGC vs. feed crude protein (*Adj. R^2^ 0.211, p<0.05*) revealed good growth in crayfish above 31% feed crude protein and protein retention of at least >14% of dietary intake (Fig S4, S5). Model of TGC vs. lipid retention (*Adj. R^2^ 0.645, p<0.05*) show a nominal lipid retention above 5% of dietary intake ensures good growth in crayfish (Fig S6). Breaching these limits may apparently lead to deteriorated growth in crayfish. The IR of ash, protein and lipid retentions in crayfish fed on different feedstuffs were estimated at 1.5-41.5%, 7.4-22.8% and 1.5-8.6% of dietary intakes, respectively. To the best of our knowledge, these are the first such quantifications on crayfish’s nutrient utilization (fed nutrient → retention → growth) following the pioneering efforts [4, 5] on different lines.


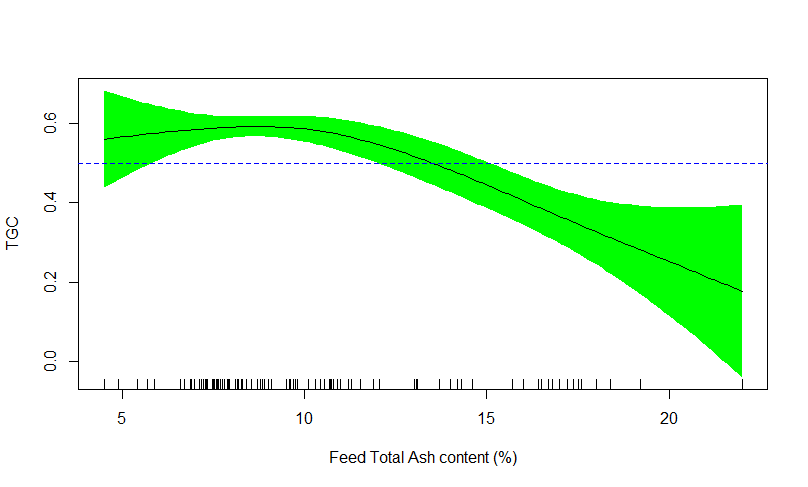


**Fig S2: Influence of feed ash content on thermal growth coefficient (TGC) of crayfish.** Horizontal dashed line marks reasonably good growth in crayfish. With increasing feed ash levels above 14%, the growth deteriorates.


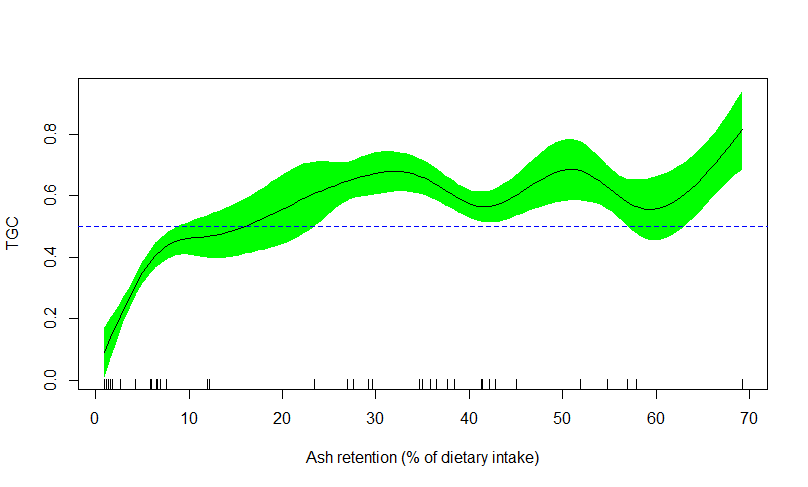


**Fig S3: Influence of dietary ash retention on thermal growth coefficient (TGC) of crayfish.** Horizontal dashed line marks reasonably good growth in crayfish. Good growth is achieved at ≥16% ash retention.


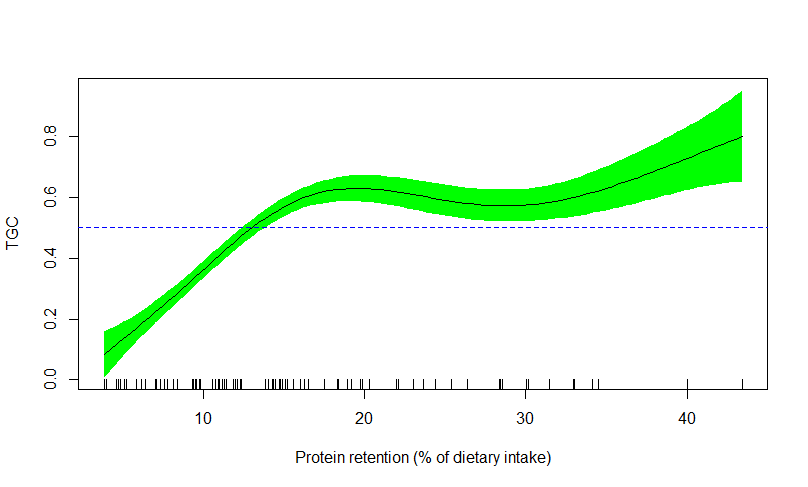


**Fig S4: Influence of dietary protein retention on thermal growth coefficient (TGC) of crayfish.** Horizontal dashed line marks reasonably good growth in crayfish. Good growth is achieved at ≥14% protein retention.


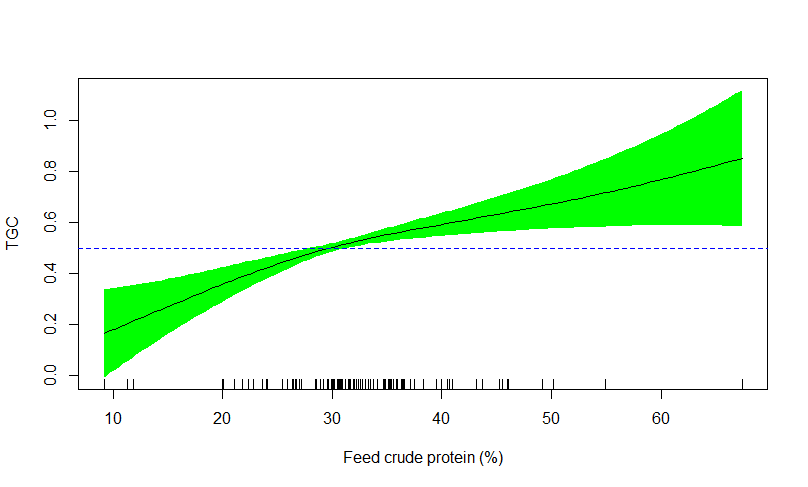


**Fig S5: Influence of feed crude protein on thermal growth coefficient (TGC) of crayfish.** Horizontal dashed line marks reasonably good growth in crayfish. With increasing crude protein (CP), the growth increases. Reasonably good growth is achieved at ≥31% CP.


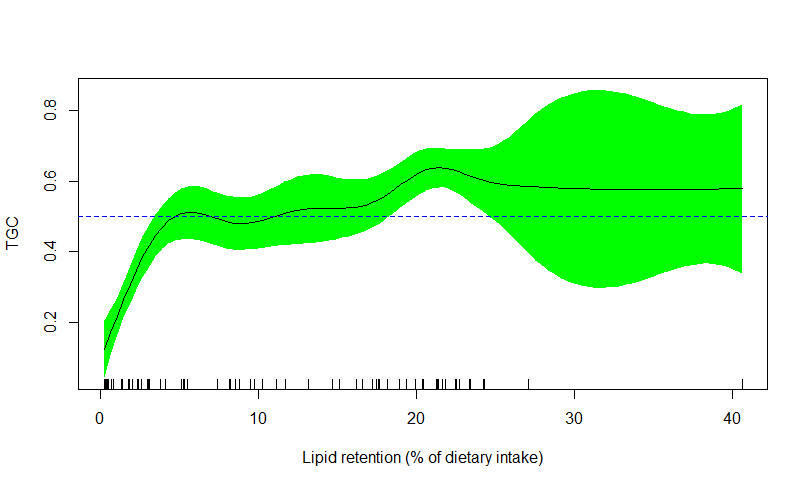


**Fig S6: Influence of dietary lipid retention on thermal growth coefficient (TGC) of crayfish.** Horizontal dashed line marks reasonably good growth in crayfish. A nominal ≥5% lipid retention seems necessary in achieving good growth.

**Supplementary observation**

Observations on pigmentation were beyond the purview of the present study. The following observations are presented for future references and further research in this regard. In groups control and BM_33_, few azure blue variants developed over time among the larger individuals (Fig S7, left panel); in contrast to individuals with normal appearance (Fig S7, right panel). Either, it may be attributed to limited carotenoid in these diets, which leads to replacement of normal astaxanthin in the exoskeleton by a protein-conjugated carotenoid called crustacyanin (azure blue color) after moltings [14]. Or, it may be a natural occurrence *i.e.* rare blue mutants with recessive French blue-body color allele [15]. Nonetheless, astaxanthin supplements in most crayfish diets are recommended (200-400 mg kg^−1^) [16, 17].


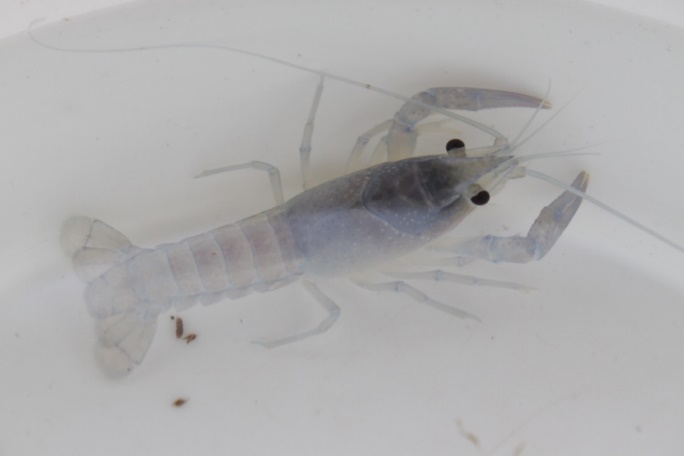

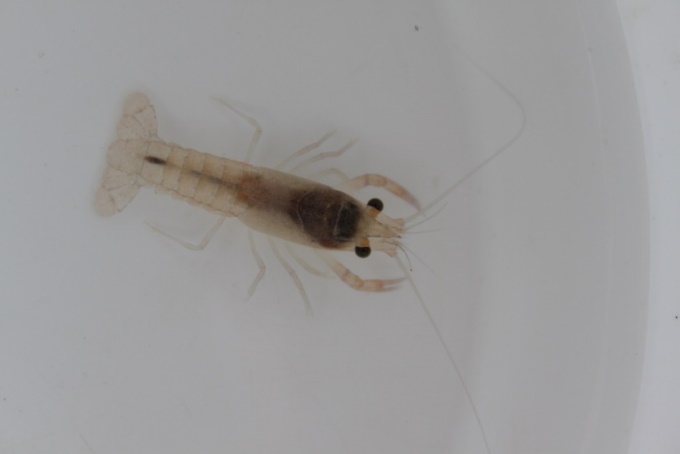


**Fig S7: *Left panel =* Rare azure blue variants of red swamp crayfish *Procambarus clarkii*.** In groups fed control (commercial feed, Coppens TILAPICO 3 mm) and BM33 (commercial feed supplemented with 33% biofloc meal), few azure blue variants developed over time among the larger individuals. ***Right panel* = A normally appearing juvenile red swamp crayfish *Procambarus clarkii* from our experimental cohort.** The observations are presented here for future references only. Photo courtesy: From experiment, the authors.

**Methods**

***Formulation of crayfish nutritional standards***

In the absence of centralized nutrition recommendations for freshwater crayfish species unlike other commercially important crustaceans (e.g. penaeid shrimps, *see* NRC [4]), available literature (growth trials on crayfish with varying protein, lipid, energy levels) were scanned. Data on proximate composition of ‘recommended’ diet profiles and their optimum, sub-optimum treatments (=nutritional profiles) were collected from 19 published studies [2, 3, 8, 9, 11, 14, 16, 18-29]. The protein-energy ratios were manually calculated.

Due to the lack of EAA requirement studies on crayfish [2], whole body amino acid compositions of *P. clarkii* (% live weight basis) reported in the literature were assumed as requirement [1, 30, 31]; a strategy common for data deficient aquatic animals [2, 4]. Attempts to standardize the mineral requirement in crayfish is virtually non-existent till date [1, 2], partly because crustaceans majorly exchange minerals with the water during ecdysis, post-molt and inter-molt stages [1]. As such, muscle and whole-body composition data of minerals (per kg live weight basis) in wild *P. clarkii* was assumed as its dietary requirement from 5 published studies [32-36]. From the compiled data, inter-quartile ranges (IRs) or median were calculated using ‘summary’ function in R [55], and proposed as the ‘crayfish standard’.

***Calculation of growth trajectory and feed utilization parameters***

From 19 published growth trials on crayfish [2, 3, 8, 9, 11, 14, 16, 18-29], thermal growth coefficient (TGC) was recalculated within a wide size range of animals (0.18-43.7 g) using the formula in Powell et al. [12]. ‘Reasonably good growth’ in crayfish was demarcated by identifying the semi-upper interquartile range (IR) of TGC from the compiled meta-dataset [37]. Growth stanzas (or, phases of growth with different rates) [12] in crayfish were visually predicted by drawing imaginary straight lines on the generalized additive model (GAM) function between body weight and TGC in R. GAMs (family: Gaussian, link function: identity) were drawn using ‘mgcv package’ in R [38].

The food conversion ratio (FCR, units), protein efficiency ratio (PER, units) and survivability (%) were determined for each diet following the formulas in Cortes-Jacinto et al. [23]. Live weight gain (LWG) was calculated applying the formula, LWG = final – initial weight (in mg)/ days reared. To eliminate statistical biasedness in the data due to hierarchical size distribution in crayfish groups, other measures of central dispersion like interquartile range (IR) and median were included besides mean. The abovementioned parameters were calculated from the IR, median and mean estimates of each treatment. All graphical models were generated using ggplot2 package in R [39].

***Quantifying*** ***nutritional dependencies on crayfish growth***

Nutrient retentions (formula, NRC [4]) were calculated from the data (nutrient fed, live weight gain and wet body composition) provided in the growth trials (listed above). Nutritional dependencies on crayfish growth were evaluated using multiple linear regression (MLR) model under ANCOVA framework [40]. Using the whole meta-dataset (n=519), the exercise was carried out in R. The response variable was TGC with growth stanzas as model diagnostics factor. Feed crude protein, feed ash, feed lipid, protein retention, ash retention and lipid retention were considered as the independent variables. The key nutritional parameters affecting growth (irrespective of the growth stages) were identified, and ‘parameter vs. TGC’ GAM model(s) were generated to quantify dependencies.

**References**

1. D’Abramo L.R. & Robinson, E.H. Nutrition of crayfish. *Rev. Aquat. Sci.* **1,** 711–728 (1989).
2. Saoud, I.P., Garza De Yta, A. & Ghanawi, J. A review of nutritional biology and dietary requirements of redclaw crayfish *Cherax quadricarinatus* (von Martens 1868). *Aquac. Nutr.* **18,** 349–368 (2012).
3. Reigh, R.C. & Ellis, S.C. Utilization of animal-protein and plant-protein supplements by red swamp crayfish *Procambarus clarkii* fed formulated diets. *J. World Aquac. Soc.* **25,** 541–552 (1994).
4. NRC (National Research Council). Nutrient requirements of fish and shrimp. National Academies Press (2011). doi:10.17226/13039
5. Zhou, Q.C. et al. Dietary arginine requirement of juvenile Pacific white shrimp, *Litopenaeus vannamei*. *Aquaculture* **364–365**, 252–258 (2012).
6. Zhu, J. et al. Optimal dietary methionine requirement of red swamp crayfish (*Procambarus clarkii*). *J. Fish. Sci. China,* **2014-02**, 1 (2014).
7. Zhang, W. et al. Effect of dietary lysime levels on grow, body composition and digestive enzyme activity of red swamp crayfish (*Procambarus clarkii*). *J. Fish. Sci. China,* **20,** 402-410 (2013).
8. Hubbard, D.M., Robinson, E.H., Brown, P.B. & Daniels, W.H. Optimum ratio of dietary protein to energy for red crayfish (*Procambarus clarkii*). *Progress. Fish-Culturist* **48,** 233–237 (1986).
9. Davis, D.A. & Robinson, E.H. Estimation of the dietary lipid requirement level of the white crayfish *Procambarus acutus acutus*. *J. World Aquac. Soc.* **17**, 37–43 (1986).
10. Xu, W.N. et al. Effect of different dietary protein and lipid levels on growth performance, body composition of juvenile red swamp crayfish (*Procambarus clarkii*). *Aquac. Int.* **21**, 687–697 (2013).
11. Cortes-Jacinto, E. et al. Effect of different dietary protein and lipid levels on growth and survival of juvenile Australian redclaw crayfish, *Cherax quadricarinatus* (von Martens). *Aquac. Nutr.* **11**, 283–291 (2005).
12. Powell, C.D., Tansil, F., France, J. & Bureau, D.P. Growth trajectory analysis of Pacific whiteleg shrimp ( *Litopenaeus vannamei* ): Comparison of the specific growth rate, the thermal‐unit growth coefficient and its adaptations. *Aquac. Res.* **51,** 480–489 (2020).
13. Hartnoll, R.G. Strategies of crustacean growth. *Aust. Museum Mem.* **18**, 121–131 (1984).
14. Huner, J.V. & Meyers, S.P. Dietary protein requirements of the red crawfish, *Procambarus clarkii* (Girard) (Decapoda, Cambaridae), grown in a closed system. *Proc. World Maric. Soc.* **10**, 751–760 (1979).
15. Black, J.B. & Huner, J.V. Genetics of the red swamp crawfish, *Procambarus clarkii* (Girard): state-of-the-art. *Proc. World Maric. Soc.* **11**, 535–543 (2009).
16. Cheng, Y. & Wu, S. Effect of dietary astaxanthin on the growth performance and nonspecific immunity of red swamp crayfish *Procambarus clarkii*. *Aquaculture* **512**, 734341 (2019).
17. Tönges, S. et al. Physiological properties and tailored feeds to support aquaculture of marbled crayfish in closed systems. bioRxiv (2020). doi:10.1101/2020.02.25.964114
18. Tan, Q., Song, D., Chen, X., Xie, S. & Shu, X. Replacing fish meal with vegetable protein sources in feed for juvenile red swamp crayfish, *Procambarus clarkii* : Effects of amino acids supplementation on growth and feed utilization. *Aquac. Nutr.* **24**, 858–864 (2018).
19. Zhang, B., Shi, Z., Wang, X. & Deng, S. The effects of hairtail protein hydrolysate–Fe2+ complexes on growth and non-specific immune response of red swamp crayfish (*Procambarus clarkii*). *Aquac. Int.* **24**, 1039–1048 (2016).
20. Xiao, X. et al. Effect of dietary cornstarch levels on growth performance, enzyme activity and hepatopancreas histology of juvenile red swamp crayfish, *Procambarus clarkii* (Girard). *Aquaculture* **426–427**, 112–119 (2014).
21. Rodríguez-González, H., Villarreal, H., García-Ulloa, M. & Hernández-Llamas, A. Dietary Lipid Requirements for Optimal Egg Quality of Redclaw Crayfish*, Cherax quadricarinatus*. *J. World Aquac. Soc.* **40**, 531–539 (2009).
22. Cortes-Jacinto, E., Villarreal-Colmenares, H., Civera-Cerecedo, R. & Martinez-Cordova, R. Effect of dietary protein level on growth and survival of juvenile freshwater crayfish *Cherax quadricarinatus* (Decapoda: Parastacidae). *Aquac. Nutr*. **9**, 207–213 (2003).
23. Cortes-jacinto, E., Villarreal-colmenares, H., Civera-cerecedo, R. & Naranjo-paramo, J. Effect of dietary protein level on the growth and survival of pre-adult freshwater crayfish *Cherax quadricarinatus* (von Martens) in monosex culture. *Aquac. Res.* **35**, 71–79 (2004).
24. Thompson, K.R., Muzinic, L.A., Engler, L.S. & Webster, C.D. Evaluation of practical diets containing different protein levels, with or without fish meal, for juvenile Australian red claw crayfish (*Cherax quadricarinatus*). *Aquaculture* **244**, 241–249 (2005).
25. Carmona-Osalde, C., Olvera-Novoa, M.A. & Rodríguez-Serna, M. Effect of the protein–lipids ratio on growth and maturation of the crayfish *Procambarus (Austrocambarus) llamasi*. *Aquaculture* **250**, 692–699 (2005).
26. Jover, M., Fernández-Carmona, J., Del Rı́o, M. & Soler, M. Effect of feeding cooked-extruded diets, containing different levels of protein, lipid and carbohydrate on growth of red swamp crayfish (*Procambarus clarkii*). *Aquaculture* **178**, 127–137 (1999).
27. Seals, C., Eversole, A.G., Tomasso, J.R. & Petrosky, B.R. Effects of temperature on feeding activity of the white river crayfish *Procambarus acutus acutus*. *J. World Aquac. Soc.* **28**, 133–141 (1997).
28. Reigh, R.C., Braden, S.L. & Laprarie, R.J. Substitution of soybean protein for fish protein in formulated diets for red swamp crawfish *Procambarus clarkii*. *J. World Aquac. Soc.* **24**, 329–338 (1993).
29. Lochmann, R., McClain, W.R. & Gatlin, D.M. Evaluation of practical feed formulations and dietary supplements for red swamp crayfish. *J. World Aquac. Soc.* **23,** 217–227 (1992).
30. Zaglol, N.F. & Eltadawy, F. Study on chemical quality and nutrition value of fresh water crayfish (*Procambarus clarkii*). *J. Arab. Aquac. Soc.* **4**, 1-18 (2009).
31. Amine, T., Aiad, A. & Abu El-Nile, M. Evaluation of chemical quality and nutrition value of fresh water cray fish (*Procambarus clarkii*). *J. High Inst. Public Heal*. **38**, 1–13 (2008).
32. Richert, J. C. & Sneddon, J. Determination of heavy metals in crawfish ( *Procambrus clarkii* ) by inductively coupled plasma–optical emission spectrometry: A study over the season in southwest Louisiana. *Anal. Lett.* **41**, 3198–3209 (2008).
33. Dierenfeld, E.S., Mcgraw, K.J., Fritsche, K., Briggler, J.T. & Ettling, J. Nutrient composition of whole crayfish (*Orconectes* and *Procambarus* Species) consumed by hellbender (*Cryptobranchus alleganiensis*). *Herpetol. Rev.* (2009).
34. Mona, M., Geasa, N. & Sharshar, K. Chemical composition of freshwater crayfish (*Procambarus clarkii*) and its nutritive value. *Egypt. J. Aquat. Biol. Fish.* **4**, 19-34 (2000).
35. Kuklina, I. et al. Accumulation of heavy metals in crayfish and fish from selected czech reservoirs. *Biomed Res. Int.* **2014**, 1–9 (2014).
36. Wen-Bin, L. Nutritional requirement and compound feed application for red swamp crayfish (*Procambarus clarkii*). *J. Econ. Anim*., **17**, 1-11 (2013).
37. Roy, K., Vrba, J., Kaushik, S.J. & Mraz, J. Feed‐based common carp farming and eutrophication: is there a reason for concern? *Rev. Aquac.* **12**, 1736-1758 (2020).
38. Wood, S. N. Generalized Additive Models. Generalized Additive Models: An Introduction with R, Second Edition. Chapman and Hall/CRC press (2017). doi:10.1201/9781315370279
39. Wickham, H. ggplot2: Elegant Graphics for Data Analysis. Springer‐Verlag, New York, NY (2016).
40. Sarkar, U.K. et al. Minnows may be more reproductively resilient to climatic variability than anticipated: Synthesis from a reproductive vulnerability assessment of Gangetic pool barbs (*Puntius sophore*). *Ecol. Indic.* **105**, 727–736 (2019).
